# Polysomnographic characteristics of severe obstructive sleep apnea vary significantly between hypertensive and normotensive patients of both genders

**DOI:** 10.1007/s11325-020-02047-8

**Published:** 2020-04-05

**Authors:** T. Leppänen, A. Kulkas, J. Töyräs, S. Myllymaa, N. Gadoth, A. Oksenberg

**Affiliations:** 1 Department of Applied Physics, University of Eastern Finland, P.O. Box 1627 (Canthia), FI-70211 Kuopio, Finland; 2 Diagnostic Imaging Center, Kuopio University Hospital, Kuopio, Finland; 3 Department of Clinical Neurophysiology, Seinäjoki Central Hospital, Seinäjoki, Finland; 4 School of Information Technology and Electrical Engineering, The University of Queensland, Brisbane, Australia; 5 Sleep Disorders Unit, Loewenstein Hospital–Rehabilitation Center, Raanana, Israel; 6 Sackler Faculty of Medicine, Tel Aviv University, Tel Aviv, Israel

**Keywords:** Apnea duration, Hypopnea duration, Phenotype, Hypertension, Gender, OSA

## Abstract

**Purpose:**

Hypertension is a common finding in patients with obstructive sleep apnea (OSA), but it has remained unclear whether or not the amount of disturbed breathing and characteristics of individual respiratory events differ between hypertensive and normotensive patients with severe OSA.

**Methods:**

Full polysomnographic recordings of 323 men and 89 women with severe OSA were analyzed. Differences in the duration of individual respiratory events, total apnea and hypopnea times, and the percentage of disturbed breathing from total sleep time (AHT%) were compared between normotensive and hypertensive patients separately by genders. Furthermore, differences in the respiratory event characteristics were assessed between three AHT% groups (AHT% ≤ 30%, 30% < AHT% ≤ 45%, and AHT% > 45%).

**Results:**

Hypertensive women had lower percentage apnea time (15.2% vs. 18.2%, *p* = 0.003) and AHT% (33.5% vs. 36.5%, *p* = 0.021) when compared with normotensive women. However, these differences were not observed between hypertensive and normotensive men. Percentage hypopnea time was higher in hypertensive men (13.5% vs. 11.2%, *p* = 0.043) but not in women (15.2% vs. 12.2%, *p* = 0.130) compared with their normotensive counterparts. The variation in AHI explained 60.5% (*ρ =* 0.778) and 65.0% (*ρ =* 0.806) of the variation in AHT% in normotensive and hypertensive patients, respectively. However, when AHT% increased, the capability of AHI to explain the variation in AHT% declined.

**Conclusions:**

There is a major inter- and intra-gender variation in percentage apnea and hypopnea times between hypertensive and normotensive patients with severe OSA. OSA is an important risk factor for hypertension and thus, early detection and phenotyping of OSA would allow timely treatment of patients with the highest risk of hypertension.

## Introduction

Obstructive sleep apnea (OSA) is a heterogeneous nocturnal breathing disorder with significant public health consequences [1]. OSA is characterized by partial (hypopneas) and complete (apneas) breathing cessations, usually leading to arousals resulting in sleep fragmentation and excessive daytime sleepiness [2] as well as recurrent but transient declines in blood oxygen saturation [3]. Due to these facts, OSA is an established risk factor for cardiovascular diseases, hypertension, and even mortality [4].

It is well established that moderate-to-severe OSA is an important risk factor for hypertension and is associated with severe health consequences such as stroke, myocardial infarction, and coronary and cerebrovascular mortality [5–8]. However, the current OSA severity classification based on the apnea-hypopnea index (AHI) is not the most reliable way to estimate the risk of OSA-related severe health consequences [9, 10]. The heterogeneity of specific OSA characteristics, such as event duration, could be one factor explaining why some patients, but not others, experience severe OSA-related health outcomes and why there are differences in the effectiveness of treatments between individual patients with severe OSA. For example, the treatment response of moderate-to-severe OSA patients to continuous positive airway pressure (CPAP) is not always optimal as CPAP does not always decrease the risk of cardiovascular events [11]. Therefore, a more accurate phenotyping of OSA patients could help to identify those patients who would gain the greatest benefits from different treatments. However, the reliable phenotyping of OSA patients is complicated due to the fact that the OSA severity grading is based almost solely on the AHI which is simply an hourly average of the number of apneas and hypopneas during sleep [12]. A more detailed disease characterization, e.g., taking into account the durations of apneas and hypopneas, could improve the estimation of risk of OSA-related health consequences [13, 14] and the identification of those individuals who would gain the greatest benefits from different therapies.

Currently, the gold standard method for OSA diagnostics is polysomnography (PSG), in which a plethora of physiological signals are recorded. However, only a small portion of the information recorded is used to assess the severity of OSA; i.e., the diagnosis relies almost exclusively on the AHI. Furthermore, based on the AHI, the severity of OSA is classified into four arbitrarily defined categories (AHI < 5, non-OSA; 5 ≤ AHI < 15, mild OSA; 15 ≤ AHI < 30, moderate OSA; AHI ≥ 30, severe OSA) [12, 15]. Nonetheless, it has been reported that this classification might not be optimal for estimating the risk of OSA-related all-cause mortality [16]. Furthermore, it has been postulated that assessing additional physiological characteristics derived from the PSG could be beneficial in phenotyping OSA in a patient-specific manner [17, 18]. For example, recently, it was shown that within the same OSA severity category, the patients can display very different disease phenotypes (e.g., there is extensive variation in the prevalence of periodic limb movements during sleep as well as in the incidence of arousals) [9]. Zinchuk et al. proposed that these differences may contribute to the risk of cardiovascular events and affect the effectiveness of treatments [9]. In fact, in that report, the AHI was not associated with the risk of cardiovascular events [9]. Thus, concerted efforts are required to examine different polysomnographic phenotypes of OSA (e.g., durations of breathing cessations and percentage time of disturbed breathing) and to clarify their effects on disease progression and treatment response.

Previously, longer respiratory events have been linked to an increased risk for all-cause mortality and cardiovascular events in patients with OSA [10, 13]. However, it is not known whether the phenotype of OSA and severity of individual respiratory events differ between hypertensive and normotensive patients with severe OSA. The working hypothesis of the present study was that regardless of gender, the phenotype of severe OSA differs and respiratory events are longer in hypertensive patients in comparison to normotensive patients. Therefore, the aim of this study was to investigate separately for both genders whether there are differences between hypertensive and normotensive patients with severe OSA in the duration of individual respiratory events, total apnea and hypopnea times, and the percentage of disturbed breathing from total sleep time.

## Patients and methods

The analysis involved 412 consecutive patients with severe OSA. The patients underwent a full PSG recording in the Sleep Disorders Unit, Loewenstein Hospital–Rehabilitation Center (Raanana, Israel) between March 2015 and October 2016. PSGs were performed using Embla S4500 devices (Natus Medical Incorporated, USA) and the setup consisted of EEG registration (C3, C4, F3, F4, O1, and O2), left and right EOG (E1-M2 and E2-M1), chin EMG (anterior, left, and right positioning), airflow (oronasal thermistor and nasal pressure), blood oxygen saturation, ECG, tibialis EMG in both legs, breathing efforts (abdomen and thorax), sleeping position, and audio and video recording of the entire night. Recordings were analyzed using RemLogic software (version 3.2, Embla Systems LCC, USA) by trained technicians in accordance with the scoring rules defined by the American Academy of Sleep Medicine in 2007 [19]. The state of the patient’s blood pressure and treatment was obtained from medical referral notes and was confirmed by the patient during the initial screening interview prior to PSG.

The durations of individual obstructive, central, and mixed apneas and hypopneas were calculated in addition to total apnea time, total hypopnea time, and total apnea+hypopnea time for each patient. In addition, the percentage of total apnea time from total sleep time (AT%), the percentage of total hypopnea time from total sleep time (HT%), and the percentage of total apnea+hypopnea time from total sleep time (AHT%) were calculated. Furthermore, the patients were divided into three different categories based on the AHT% histogram (AHT% ≤ 30%, 30% < AHT% ≤ 45%, and AHT% > 45%) having almost equal numbers of patients in each category. The Mann-Whitney U test was used to evaluate the statistical significance of differences in demographic data, polysomnographic data, and the Epworth Sleepiness Scale (ESS) between (1) different AHT% categories, (2) between genders, and (3) between normotensive (i.e., patients with no antihypertensive medication) and hypertensive patients (i.e., patients using antihypertensive medication). The Chi-square test was used to assess the statistical significance of differences in the prevalence of hypertension between the different AHT% categories and between genders. Furthermore, the evaluation of the differences in the total apnea time, total hypopnea time, total apnea+hypopnea time, AT%, HT%, AHT%, and durations of obstructive apneas, central apneas, mixed apneas, and hypopneas between different pools of patients was done with analysis of covariance (ANCOVA) adjusted for age, body mass index (BMI), and AHI. The association between the AHI and the AHT% was assessed with the Spearman correlation (*ρ*). Statistical analyses were performed with SPPS (version 24, SPSS Inc., USA) and *p* < 0.05 was considered as the limit for statistical significance. *p*_MWU_, *p*_Cs_, and *p*_A_ denote *p* values obtained from the Mann-Whitney U test, Chi-square test, and ANCOVA, respectively. *p*_Spea_ denotes the statistical significance of the Spearman correlation.

## Results

The studied cohort consisted only of patients diagnosed with severe OSA (median AHI 50.6 events/h). The majority of patients were males (78.4%), elderly (median age 60.9 years), and obese (median BMI 32.3 kg/m^2^). Hypertensive patients were, in general, older (*p*_MWU_ < 0.001) and had a higher BMI (*p*_MWU_ = 0.025) than their normotensive counterparts (Table 1). Even though no statistically significant differences were seen in the AHI or oxygen desaturation index (ODI), hypertensive patients had longer total hypopnea times (44.4 min vs. 35.6 min, *p*_MWU_ = 0.020, *p*_A_ = 0.027) and higher HT% (14.2% vs. 11.3%, *p*_MWU_ = 0.003, *p*_A_ = 0.031) when compared with normotensive patients (Table 1). There were no statistically significant differences in the duration of respiratory events (*p*_MWU_ ≥ 0.223, *p*_A_ ≥ 0.056) or in the ESS score (*p*_MWU_ = 0.302) between hypertensive and normotensive patients (Table 1).

**Table 1:** Demographic and polysomnographic data (median (interquartile range)) in normotensive and hypertensive patients having severe OSA

	Normotensive	Hypertensive	*p*_MWU_ and *p*_Cs_ values*	*p*_A_ value^#^
Patients (*n*)	219	193	-	-
Male (*n* (%))	174 (79.5)	149 (77.2)	0.580	-
Female (*n* (%))	45 (20.5)	44 (22.8)		
Age (years)	55.8 (43.3–65.0)	65.3 (56.5–72.4)	< 0.001	-
BMI (kg/m^2^)	32.0 (28.7–36.4)	33.3 (29.7–37.7)	0.025	-
TST total (min)	356 (304–399)	331 (292–379)	0.004	-
TST supine (min)	123 (45–222)	95 (25–167)	0.002	-
TST lateral (min)	194 (99–260)	209 (139–267)	0.028	-
TST REM (min)	58 (37–86)	54 (34–72)	0.014	-
TST NREM (min)	293 (249–323)	282 (236–318)	0.125	-
AHI total (1/h)	50.4 (38.1–68.3)	50.7 (39.9–68.0)	0.448	-
AHI supine (1/h)	67.5 (47.7–85.0)	66.3 (51.4–83.2)	0.543	-
AHI lateral (1/h)	42.9 (21.1–69.2)	52.7 (31.3–79.9)	0.006	-
AHI REM (1/h)	56.0 (41.1–68.2)	51.3 (39.6–66.8)	0.385	-
AHI NREM (1/h)	52.6 (35.5–68.6)	48.5 (39.7–69.6)	0.127	-
ODI total (1/h)	42.6 (27.0–57.6)	38.1 (29.3–62.1)	0.118	-
ODI REM (1/h)	51.0 (28.7–62.6)	40.0 (30.5–64.6)	0.527	-
ODI NREM (1/h)	42.4 (24.8–58.3)	39.7 (28.4–62.7)	0.053	-
Arousal index (1/h)	57.8 (44.3–72.0)	57.1 (47.3–75.1)	0.130	-
Total apnea time (min)	78.4 (39.9–149.5)	71.5 (35.0–121.6)	0.068	0.035
Total hypopnea time (min)	35.6 (17.3–56.3)	44.4 (24.8–61.1)	0.020	0.027
Total apnea+hypopnea time (min)	126.7 (88.9–190.2)	124.1 (89.7–161.6)	0.340	0.298
AT% (% from TST)	23.2 (12.4–43.1)	22.3 (11.0–34.6)	0.228	0.024
HT% (% from TST)	11.3 (5.2–16.5)	14.2 (7.8–18.8)	0.003	0.031
AHT% (% from TST)	37.9 (26.8–54.6)	37.9 (29.4–49.2)	0.869	0.236
Obstructive apnea duration (s)	25.0 (21.4–30.3)	24.8 (21.3–29.6)	0.605	0.247
Central apnea duration (s)	16.8 (14.5–19.6)	16.6 (13.9–19.2)	0.423	0.056
Mixed apnea duration (s)	32.5 (28.8–40.8)	33.0 (26.8–41.0)	0.891	0.572
Hypopnea duration (s)	26.6 (22.5–30.7)	25.5 (22.1–29.5)	0.223	0.504
ESS (points)	9 (5–15)	9 (5–13)	0.302	-

*BMI* body mass index, *TST* total sleep time, *REM* rapid eye movement sleep, *NREM* non-rapid eye movement sleep, *AHI* apnea-hypopnea index, *ODI* oxygen desaturation index, *AT%* percentage of total apnea time from TST, *HT%* percentage of total hypopnea time from TST, *AHT%* percentage of total apnea+hypopnea time from TST, *ESS* Epworth Sleepiness Scale

*Statistical significance of differences was evaluated with Chi-square test (*p*_Cs_ value) for categorical variables and with Mann-Whitney U test (*p*_MWU_ value) for continuous variables

^#^Analysis of covariance (ANCOVA, *p*_A_ value) was adjusted for age, body mass index, and apnea-hypopnea index

Males were younger and leaner (*p*_MWU_ = 0.011) than females. Although there were no statistically significant differences in the overall AHI or ODI values, females had higher values of AHI (*p*_MWU_ = 0.002) and ODI (*p*_MWU_ = 0.016) during REM sleep (Table 2). The total apnea time (81.5 min vs. 55.9 min, *p*_MWU_ = 0.002, *p*_A_ < 0.001) was longer in males while the total hypopnea time (39.5 min vs. 45.1 min, *p*_MWU_ = 0.023, *p*_A_ = 0.014) was longer in females (Table 2), resulting in higher AHT% in males (38.8% vs. 35.4%, *p*_MWU_ = 0.011, *p*_A_ < 0.001). Furthermore, males had longer obstructive apneas (*p*_MWU_ < 0.001, *p*_A_ < 0.001), central apneas (*p*_MWU_ = 0.012, *p*_A_ = 0.009), and hypopneas (*p*_MWU_ = 0.007, *p*_A_ = 0.032). Instead, no statistically significant differences between genders were observed in the ESS score or in the prevalence of hypertension (*p*_MWU_ ≥ 0.580, Table 2).

**Table 2:** Demographic and polysomnographic data (median (interquartile range)) in male and female patients having severe OSA

	Male	Female	*p*_MWU_ and *p*_Cs_ values*	*p*_A_ value^#^
Patients (*n*)	323	89	-	-
Age (years)	60.4 (47.2–68.8)	64.0 (56.5–70.6)	0.011	-
BMI (kg/m^2^)	32.2 (29.4–36.4)	34.6 (29.8–40.5)	0.011	-
TST total (min)	348 (299–392)	333 (294–378)	0.224	-
TST supine (min)	112 (42–203)	89 (26–191)	0.299	-
TST lateral (min)	200 (123–263)	205 (122–272)	0.616	-
TST REM (min)	57 (34–80)	55 (38–79)	0.927	-
TST NREM (min)	289 (243–322)	276 (237–311)	0.229	-
AHI total (1/h)	49.9 (39.2–68.3)	51.2 (37.8–66.1)	0.804	-
AHI supine (1/h)	67.1 (50.4–84.6)	65.2 (47.5–80.3)	0.431	-
AHI lateral (1/h)	46.4 (26.7–72.0)	48.3 (25.9–78.2)	0.633	-
AHI REM (1/h)	53.7 (39.0–66.1)	60.0 (48.7–74.1)	0.002	-
AHI NREM (1/h)	50.3 (38.4–69.5)	47.8 (34.4–64.1)	0.248	-
ODI total (1/h)	40.2 (28.4–61.1)	41.6 (26.9–55.2)	0.599	-
ODI REM (1/h)	46.2 (28.4–62.6)	52.1 (41.2–69.7)	0.016	-
ODI NREM (1/h)	41.0 (27.8–61.8)	39.4 (24.1–55.0)	0.208	-
Arousal index (1/h)	59.3 (46.1–73.9)	55.7 (45.3–71.3)	0.508	-
Total apnea time (min)	81.5 (40.3–140.1)	55.9 (33.1–96.2)	0.002	< 0.001
Total hypopnea time (min)	39.5 (19.2–58.3)	45.1 (27.7–64.9)	0.023	0.014
Total apnea+hypopnea time (min)	127.8 (92.9–184.1)	108.3 (79.4–158.0)	0.014	0.008
AT% (% from TST)	24.7 (12.7–43.4)	17.7 (10.1–29.1)	0.001	< 0.001
HT% (% from TST)	11.8 (6.4–17.6)	13.8 (9.1–20.0)	0.018	0.022
AHT% (% from TST)	38.8 (28.9–52.8)	35.4 (25.1–44.7)	0.011	< 0.001
Obstructive apnea duration (s)	25.3 (22.4–30.5)	22.7 (19.1–27.5)	< 0.001	< 0.001
Central apnea duration (s)	17.3 (14.6–19.6)	15.3 (12.8–17.9)	0.012	0.009
Mixed apnea duration (s)	32.8 (28.5–41.3)	30.6 (23.8–38.6)	0.114	0.021
Hypopnea duration (s)	26.5 (22.7–30.7)	24.5 (20.3–27.7)	0.007	0.032
ESS (points)	9 (5–14)	9 (5–14)	0.668	-
Hypertension (*n* (%))	149 (46.1)	44 (49.4)	0.580	-

*BMI* body mass index, *TST* total sleep time, *REM* rapid eye movement sleep, *NREM* non-rapid eye movement sleep, *AHI* apnea-hypopnea index, *ODI* oxygen desaturation index, *AT*% percentage of total apnea time from TST, *HT%* percentage of total hypopnea time from TST, *AHT%* percentage of total apnea+hypopnea time from TST, *ESS* Epworth Sleepiness Scale

*Statistical significance of differences was evaluated with Chi-square test (*p*_Cs_ value) for categorical variables and with Mann-Whitney U test (*p*_MWU_ value) for continuous variables

^#^Analysis of covariance (ANCOVA, *p*_A_ value) was adjusted for age, body mass index, and apnea-hypopnea index

The females with hypertension were on average 10 years older (*p*_MWU_ < 0.001) than normotensive female patients (Table 3). Normotensive female patients had higher values of total sleep time (TST) (353 min vs. 323 min, *p*_MWU_ = 0.028) possibly partly explaining the longer observed total apnea time (61.4 min vs. 42.8 min, *p*_MWU_ = 0.014, *p*_A_ = 0.007) in comparison with hypertensive female patients. Furthermore, the ANCOVA test showed that the AHT% was significantly (*p*_A_ = 0.021) higher in normotensive females than in the hypertensive females after adjustment for age, BMI, and AHI (Table 4). In contrast, hypertensive females tended to have a higher HT% (12.2% vs. 15.2%, *p*_MWU_ = 0.053, *p*_A_ = 0.130) but statistical significance was not reached (Table 3).

**Table 3:** Demographic and polysomnographic data (median (interquartile range)) in normotensive and hypertensive female patients having severe OSA

	Female—normotensive	Female—hypertensive	*p*_MWU_ values*	*p*_A_ value^#^
Patients (*n* (%))	45 (50.6)	44 (49.4)	-	-
Age (years)	59.2 (49.5–64.6)	69.2 (63.8–74.0)	< 0.001	-
BMI (kg/m^2^)	32.3 (29.1–39.9)	36.0 (30.5–41.4)	0.161	-
TST total (min)	353 (304–385)	323 (280–360)	0.028	-
TST supine (min)	87 (24–207)	91 (26–176)	0.501	-
TST lateral (min)	221 (103–269)	201 (129–273)	0.768	-
TST REM (min)	59 (42–85)	52 (34–74)	0.130	-
TST NREM (min)	285 (239–313)	271 (216–305)	0.188	-
AHI total (1/h)	51.2 (41.2–67.6)	51.2 (36.5–60.8)	0.580	-
AHI supine (1/h)	68.3 (51.0–80.8)	60.9 (39.4–77.5)	0.148	-
AHI lateral (1/h)	51.1 (29.8–83.1)	41.6 (21.7–67.3)	0.215	-
AHI REM (1/h)	61.5 (51.5–79.4)	58.8 (44.0–69.4)	0.113	-
AHI NREM (1/h)	47.8 (36.1–66.4)	48.1 (33.1–61.3)	0.821	-
ODI total (1/h)	44.2 (27.4–60.2)	40.1 (25.5–54.2)	0.531	-
ODI REM (1/h)	51.3 (37.5–70.4)	58.1 (41.3–67.7)	0.950	-
ODI NREM (1/h)	39.4 (24.4–58.6)	38.4 (22.3–54.6)	0.733	-
Arousal index (1/h)	55.2 (45.2–65.3)	57.0 (46.6–75.1)	0.565	-
Total apnea time (min)	61.4 (36.2–129.7)	42.8 (24.2–78.3)	0.014	0.007
Total hypopnea time (min)	39.6 (17.6–72.4)	47.9 (33.5–64.3)	0.162	0.124
Total apnea+hypopnea time (min)	131.3 (84.2–181.3)	100.8 (74.2–137.3)	0.017	0.069
AT% (% from TST)	18.2 (11.6–33.9)	15.2 (8.3–25.8)	0.067	0.003
HT% (% from TST)	12.2 (5.2–20.7)	15.2 (10.7–19.4)	0.053	0.130
AHT% (% from TST)	36.5 (26.3–54.2)	33.5 (22.8–42.0)	0.146	0.021
Obstructive apnea duration (s)	24.0 (19.5–30.1)	21.3 (18.8–25.6)	0.076	0.095
Central apnea duration (s)	15.6 (12.9–19.1)	15.0 (12.7–17.5)	0.512	0.040
Mixed apnea duration (s)	36.2 (30.5–40.0)	25.3 (22.2–31.1)	0.072	0.049
Hypopnea duration (s)	26.3 (20.5–30.2)	23.6 (20.0–26.3)	0.069	0.213
ESS (points)	11 (5–17)	9 (6–13)	0.377	-

*BMI* body mass index, *TST* total sleep time, *REM* rapid eye movement sleep, *NREM* non-rapid eye movement sleep, *AHI* apnea-hypopnea index, *ODI* oxygen desaturation index, *AT%* percentage of total apnea time from TST, *HT%* percentage of total hypopnea time from TST, *AHT%* percentage of total apnea+hypopnea time from TST, *ESS* Epworth Sleepiness Scale

*Statistical significance of differences was evaluated with Mann-Whitney U test (*p*_MWU_ value)

^#^Analysis of covariance (ANCOVA, *p*_A_ value) was adjusted for age, body mass index, and apnea-hypopnea index

**Table 4:** Evaluation of the difference in the percentage of total apnea+hypopnea time from total sleep time (AHT%) between normotensive and hypertensive female and male patients having severe OSA. Adjusted AHT% means were 39.1 and 34.5 for normotensive and hypertensive female patients, respectively. Adjusted AHT% means were 42.0 and 41.2 for normotensive and hypertensive male patients, respectively

	*B*	SD error	*p*_A_ value	95% CI (lower–upper)	
Females					
Age (years)	0.136	0.081	0.097	− 0.025	0.298
BMI (kg/m^2^)	− 0.357	0.119	0.003	− 0.593	− 0.121
AHI total (1/h)	0.525	0.038	< 0.001	0.449	0.601
Hypertension*	4.568	1.946	0.021	0.698	8.437
Males					
Age (years)	0.061	0.041	0.138	− 0.020	0.142
BMI (kg/m^2^)	− 0.062	0.105	0.553	− 0.268	0.144
AHI total (1/h)	0.622	0.031	< 0.001	0.561	0.684
Hypertension*	0.789	1.190	0.508	− 1.552	3.130

*B* a partial regression coefficient, *SD error* variation of the partial regression coefficient, *CI* confidence interval. Analysis of covariance (ANCOVA, *p*_A_ value) was adjusted for age, body mass index, and apnea-hypopnea index

*Hypertensive patients were used as the reference

The hypertensive males were older than the normotensive males (64.6 years vs. 54.7 years, *p*_MWU_ < 0.001, Table 5). Normotensive males had higher TST values (360 min vs. 339 min, *p*_MWU_ = 0.025) compared with hypertensive males but no significant differences were observed in total apnea time (*p*_MWU_ = 0.450, *p*_A_ = 0.100). Although the adjusted AHT% did not differ statistically significantly (*p*_A_ = 0.508) between hypertensive and normotensive males (Table 4), hypertensive males had statistically significantly higher HT% than their normotensive counterparts (13.5% vs. 11.2%, *p*_MWU_ = 0.028, *p*_A_ = 0.043, Table 5). No statistically significant differences were found in respiratory event durations (*p*_MWU_ ≥ 0.537, *p*_A_ ≥ 0.251) between normotensive and hypertensive male patients (Table 5).

**Table 5:** Demographic and polysomnographic data (median (interquartile range)) in normotensive and hypertensive male patients having severe OSA

	Male—normotensive	Male—hypertensive	*p*_MWU_ values*	*p*_A_ value^#^
Patients (*n* (%))	174 (53.9)	149 (46.1)	-	-
Age (years)	54.7 (42.2–65.1)	64.6 (54.6–71.8)	< 0.001	-
BMI (kg/m^2^)	31.8 (28.6–35.8)	32.5 (29.6–36.9)	0.089	-
TST total (min)	360 (304–404)	339 (296–381)	0.025	-
TST supine (min)	135 (51–227)	99 (24–167)	0.003	-
TST lateral (min)	188 (92–258)	210 (143–266)	0.017	-
TST REM (min)	57 (35–87)	55 (34–72)	0.041	-
TST NREM (min)	293 (252–326)	285 (238–319)	0.280	-
AHI total (1/h)	49.0 (37.0–68.4)	50.7 (40.7–68.1)	0.217	-
AHI supine (1/h)	67.0 (46.2–85.5)	68.2 (52.8–83.9)	0.968	-
AHI lateral (1/h)	41.4 (20.1–64.3)	58.3 (31.6–81.3)	< 0.001	-
AHI REM (1/h)	54.3 (37.5–65.0)	53.1 (39.3–66.1)	0.830	-
AHI NREM (1/h)	48.9 (35.4–68.7)	52.1 (40.9–70.3)	0.054	-
ODI total (1/h)	38.0 (26.7–57.4)	45.8 (29.7–64.2)	0.038	-
ODI REM (1/h)	46.5 (28.1–61.6)	46.0 (28.6–63.9)	0.584	-
ODI NREM (1/h)	36.7 (25.4–58.6)	45.5 (29.4–66.4)	0.014	-
Arousal index (1/h)	58.6 (43.7–73.1)	60.8 (47.6–75.4)	0.153	-
Total apnea time (min)	84.0 (40.9–161.3)	79.2 (37.1–130.9)	0.450	0.100
Total hypopnea time (min)	33.9 (16.5–55.2)	41.3 (21.7–60.9)	0.076	0.032
Total apnea+hypopnea time (min)	125.5 (90.3–192.5)	130.4 (97.1–176.8)	0.843	0.568
AT% (% from TST)	23.7 (12.7–44.1)	24.9 (13.9–41.1)	0.732	0.077
HT% (% from TST)	11.2 (5.1–16.3)	13.5 (7.0–18.5)	0.028	0.043
AHT% (% from TST)	38.5 (26.8–54.7)	39.8 (30.3–52.0)	0.323	0.508
Obstructive apnea duration (s)	25.0 (21.7–30.4)	25.7 (22.6–30.6)	0.615	0.637
Central apnea duration (s)	17.3 (14.8–19.8)	17.5 (14.0–19.5)	0.707	0.251
Mixed apnea duration (s)	32.4 (28.6–41.1)	34.0 (28.1–42.2)	0.537	0.994
Hypopnea duration (s)	26.8 (22.6–30.9)	26.3 (22.9–30.3)	0.720	0.874
ESS (points)	9 (5–15)	8 (5–14)	0.468	-

*BMI* body mass index, *TST* total sleep time, *REM* rapid eye movement sleep, *NREM* non-rapid eye movement sleep, *AHI* apnea-hypopnea index, *ODI* oxygen desaturation index, *AT%* percentage of total apnea time from TST, *HT*% percentage of total hypopnea time from TST, *AHT*% percentage of total apnea+hypopnea time from TST, *ESS* Epworth Sleepiness Scale

*Statistical significance of differences was evaluated with Mann-Whitney U test (*p*_MWU_ value)

^#^Analysis of covariance (ANCOVA, *p*_A_ value) was adjusted for age, body mass index, and apnea-hypopnea index

**Fig. 1:**
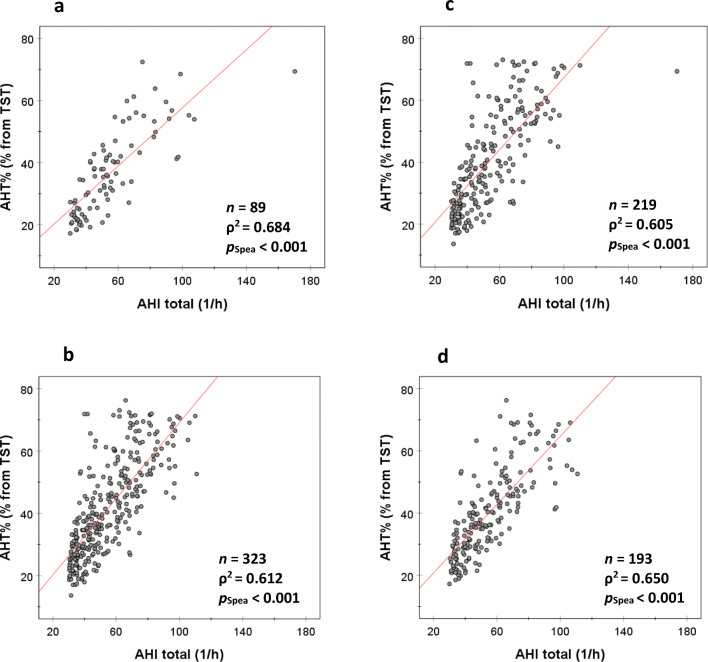
There is an increase in the percentage time of total apnea+hypopnea time from total sleep time (AHT%) with an elevation in the apnea-hypopnea index (AHI) in **a** females, **b** males, **c** normotensive patients, and **d** hypertensive patients. However, the variation in AHI explained only 68.4% (*ρ* = 0.827) and 61.2% (*ρ* = 0.782) of the variation in AHT% in females and males, respectively, and 60.5% (*ρ* = 0.778) and 65.0% (ρ = 0.806) in normotensive and hypertensive patients, respectively

**Fig. 2:**
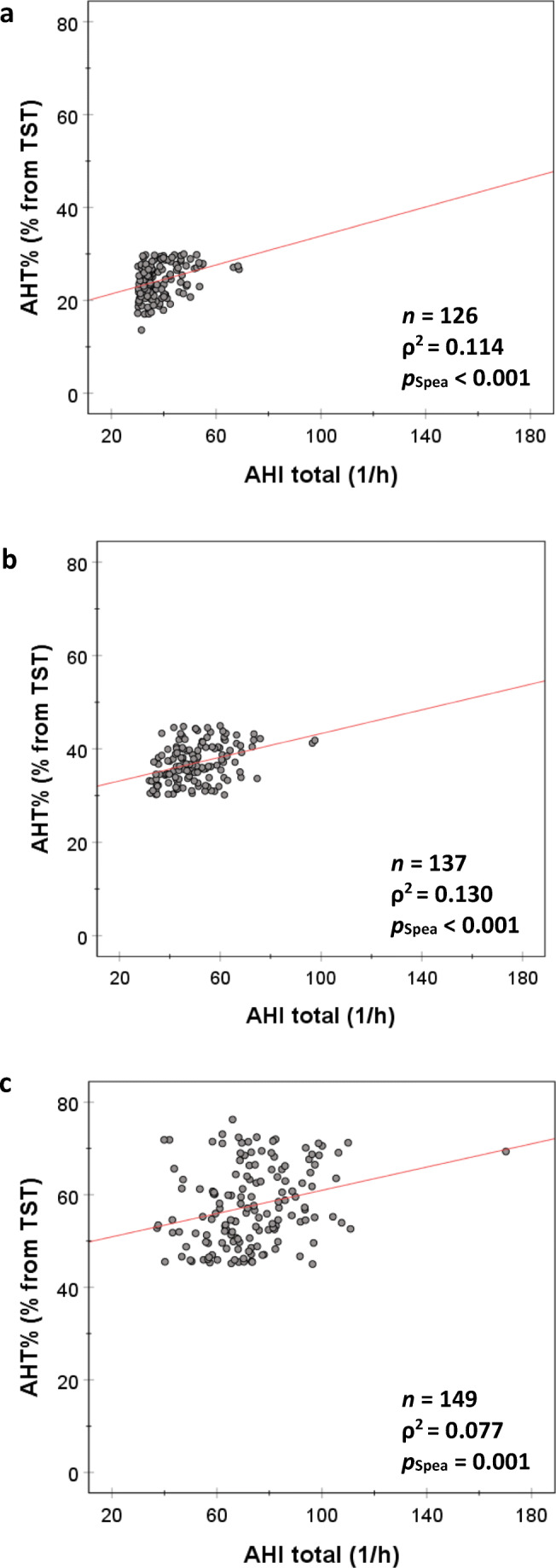
The percentage time of total apnea+hypopnea time from total sleep time (AHT%) as a function of the apnea-hypopnea index (AHI) in different AHT% categories: **a** AHT% ≤ 30%, **b** 30% < AHT% ≤ 45%, and **c** AHT% > 45%. The variation in AHI explained only 11.4% (*ρ* = 0.337), 13.0% (*ρ* = 0.360), and 7.7% (*ρ* = 0.278) of the variation in AHT% in these categories, respectively

In females and males, the AHT% increased significantly with the increase in AHI (*p*_Spea_ < 0.001, Fig. 1). The variation in AHI explained 68.4% (*ρ =* 0.827, *ρ*^2^ = 0.684) and 61.2% (*ρ =* 0.782, *ρ*^2^ = 0.612) of the variation in AHT% in females and males, respectively. The same phenomenon was observed in normotensive and hypertensive patients; i.e., 60.5% (*ρ =* 0.778, *ρ*^2^ = 0.605) and 65.0% (*ρ =* 0.806, *ρ*^2^ = 0.650) of the variation in AHT% were explained by the variation in AHI in normotensive and hypertensive patients, respectively (*p*_Spea_ < 0.001, Fig. 1). However, when AHT% increased (i.e., when severe OSA patients were divided into categories based on their AHT%), the capability of AHI to explain the increase in AHT% declined (Fig. 2). For example, in patients with AHT% > 45%, the variation in AHI explained only 7.7% (*ρ =* 0.278, *ρ*^2^ = 0.077) of the variation in AHT%. It is also notable that total apnea time increased statistically significantly (*p*_MWU_ < 0.05) towards the higher AHT% categories while total hypopnea time did not (Table 6). This may be partially explained by the increasing duration of apneas evident in the higher AHT% categories (*p*_MWU_ < 0.05), central apneas being the only exception. There were no significant differences in the duration of hypopneas between the AHT% ≤ 30% and AHT% > 45% categories.

**Table 6:** Demographic and polysomnographic data (median (interquartile range)) in different AHT% categories

	AHT% ≤ 30%	30% < AHT% ≤ 45%	AHT% > 45%
Patients (*n*)	126	137	149
Male (*n* (%))	93 (73.8)	103 (75.2)	127 (85.2)
Female (*n* (%))	33 (26.2)	34 (24.8)	22 (14.8)
Age (years)	60.8 (47.9–69.7)	63.7 (52.5–70.7)	59.8 (46.9–67.6)^#^
BMI (kg/m^2^)	31.8 (29.4–35.6)	31.8 (29.1–36.2)	33.8 (30.1–38.6)*^#^
TST total (min)	327 (292–385)	340 (299–385)	354 (305–401)*
TST supine (min)	96 (25–181)	113 (40–177)	121 (46–235)
TST lateral (min)	204 (133–263)	216 (131–269)	191 (101–258)
TST REM (min)	60 (37–83)	55 (37–79)	54 (31–78)
TST NREM (min)	273 (229–305)	283 (241–322)	301 (259–334)*
AHI total (1/h)	35.8 (32.8–42.5)	49.0 (42.5–57.8)*	72.0 (62.1–83.2)*^#^
AHI supine (1/h)	61.1 (38.9–78.1)	62.3 (49.7–75.2)	78.0 (63.9–88.5)*^#^
AHI lateral (1/h)	30.8 (15.0–46.2)	41.6 (26.7–58.3)*	70.9 (52.0–90.5)*^#^
AHI REM (1/h)	51.0 (33.4–62.2)	49.4 (39.5–63.0)	61.4 (51.7–75.0)*^#^
AHI NREM (1/h)	33.9 (28.0–40.9)	47.4 (39.9–57.6)*	73.4 (64.0–84.8)*^#^
ODI total (1/h)	26.7 (21.4–35.5)	39.4 (29.6–50.4)*	63.8 (50.8–76.8)*^#^
ODI REM (1/h)	39.7 (24.0–58.5)	43.4 (28.0–58.2)	57.8 (47.9–71.0)*^#^
ODI NREM (1/h)	24.2 (17.9–31.3)	37.1 (29.0–49.4)*	65.6 (50.6–77.3)*^#^
Arousal index (1/h)	43.7 (37.9–55.7)	55.8 (47.2–64.6)*	76.1 (64.8–88.2)*^#^
Total apnea time (min)	35.0 (21.5–49.7)	72.0 (46.4–95.2)*	167.2 (115.9–224.4)*^#^
Total hypopnea time (min)	42.0 (27.6–56.2)	46.9 (29.7–66.1)*	24.8 (7.8–56.0)*^#^
Total apnea+hypopnea time (min)	79.9 (65.1–93.9)	126.2 (107.6–142.4)*	204.5 (161.6–243.7)*^#^
AT% (% from TST)	11.1 (7.0–15.1)	22.0 (15.1–27.9)*	47.6 (34.6–60.3)*^#^
HT% (% from TST)	13.1 (9.3–16.7)	14.6 (9.1–20.5)*	6.9 (2.0–17.7)*^#^
AHT% (% from TST)	24.8 (21.4–27.4)	36.7 (33.6–40.4)*	55.9 (50.5–64.6)*^#^
Obstructive apnea duration (s)	22.5 (18.0–25.7)	25.7 (21.6–30.8)*	27.1 (23.5–32.4)*^#^
Central apnea duration (s)	15.7 (13.9–18.0)	18.1 (14.6–19.8)*	17.6 (14.3–19.7)*
Mixed apnea duration (s)	26.7 (21.7–32.7)	32.5 (26.5–37.2)*	36.4 (30.7–42.5)*^#^
Hypopnea duration (s)	24.5 (21.3–28.6)	27.5 (24.1–32.2)*	26.0 (21.7–30.3)^#^
ESS (points)	9 (5–13)	8 (5–14)	10 (6–16)*^#^
Hypertension (*n* (%))	51 (40.5)	78 (56.9)	64 (43.0)

*BMI* body mass index, *TST* total sleep time, *REM* rapid eye movement sleep, *NREM* non-rapid eye movement sleep, *AHI* apnea-hypopnea index, *ODI* oxygen desaturation index, *AT%* percentage of total apnea time from TST, *HT%* percentage of total hypopnea time from TST, *AHT%* percentage of total apnea+hypopnea time from TST, *ESS* Epworth Sleepiness Scale

*Significantly different from AHT% < 30% group (Mann-Whitney U)

^#^Significantly different from AHT% 30%–45% group (Mann-Whitney U)

Gender distribution (*p*_Cs_ = 0.038) and hypertension (*p*_Cs_ = 0.014) differed statistically significantly between AHT% groups (Chi-square test)

In general, there was no significant differences in subjectively measured daytime sleepiness between male and female patients (median ESS score, 9 for both genders, *p*_MWU_ = 0.668, Table 2) or between hypertensive and normotensive patients (median ESS score, 9 for both, *p*_MWU_ = 0.302, Table 1). In addition, the ESS score did not differ statistically significantly between hypertensive and normotensive females (*p*_MWU_ = 0.377, Table 3) or between hypertensive and normotensive males (*p*_MWU_ = 0.468, Table 5). However, those patients with AHT% > 45% had a higher arousal index (*p*_MWU_ < 0.05) and higher ESS scores (median ESS, 10) when compared with patients belonging to the lower AHT% categories (*p*_MWU_ < 0.05, Table 6).

## Discussion

The present study investigated whether the phenotype of OSA and the duration of individual respiratory events differ between hypertensive and normotensive patients with severe OSA, separately for female (*n* = 89) and male (*n* = 323) patients. We found that the hypertensive patients were older (*p*_MWU_ < 0.001) regardless of gender and that hypertensive males had higher HT% (*p*_MWU_ = 0.028, *p*_A_ = 0.043) than their normotensive counterparts. HT% tended to be higher also in hypertensive female patients (*p*_MWU_ = 0.053, *p*_A_ = 0.130) as compared with normotensive females but the limit of statistical significance was not reached. In addition, hypertensive females had a significantly lower total apnea time than normotensive females (*p*_MWU_ = 0.014, *p*_A_ = 0.007) and this difference also remained statistically significant when normalized with TST after adjusting for covariates (i.e., AT%, *p*_A_ = 0.003). The patients with AHT% > 45% were subjectively sleepier than the other patients. The variation in AHI explained only two-thirds of the variation observed in the AHT% in normotensive and hypertensive patients, regardless of gender. However, when patients were divided into AHT% categories, the variation in AHI explained only 7.7% of this variation in the patients with AHT% > 45%. This indicates that the AHI alone is not capable of explaining the patient-specific differences in characteristics of OSA and further highlights the need for more individualized estimation of OSA severity. Therefore, the severity estimation of OSA and the treatment decisions should not be based exclusively on the AHI. Taking into consideration also the duration of respiratory events and the percentage time of disturbed breathing could lead to a better optimization of individualized treatments and subsequently to a better treatment response.

In general, female patients with OSA are more obese and older than their male counterparts [20] and the present results involving only severe OSA patients are in line with that finding. Furthermore, it has been reported that females have more hypopnea-oriented disease [21], a finding confirmed in our study. In the presently studied pool of patients, males had higher values of AT% and AHT% but lower HT% than females. In addition, the respiratory events were longer in male patients. Interestingly, this was observed without significant gender differences in the overall AHI even though AHI during REM sleep was significantly higher in females. The present findings which reveal major inter-individual differences in the characteristics of OSA support the idea that the best possible treatment outcomes could be achieved by adopting individualized treatment protocols. However, no significant differences in subjective sleepiness or in the prevalence of hypertension between genders was witnessed, indicating similar prevalences of objective sleepiness and hypertension between genders in the current severe OSA population.

Hypertension has been strongly linked to OSA and the prevalence of hypertension increases with increasing severity of OSA [22, 23]. In OSA patients, it has been reported that sympathetic activity is elevated during sleep due to repeated inspiratory efforts against obstructed airways, hypoxia, and respiratory event–related arousals [22]. Moreover, heart rate and blood pressure are markedly increased from their normal levels during post-apneic periods when the patient is asleep [22]. As OSA-related cardiovascular consequences are not only confined to sleep [22], it could be speculated that daytime hypertension is a continuum from the transient nocturnal blood pressure changes. However, the present results suggest that the AHI is not the only explanatory polysomnographic factor for hypertension among severe OSA patients. Interestingly, we found that hypertensive females tended to have higher HT% (*p*_MWU_ = 0.053, *p*_A_ = 0.130) and lower AT% (*p*_MWU_ = 0.067, *p*_A_ = 0.003) than normotensive females and that their obstructive apneas (*p*_MWU_ = 0.076, *p*_A_ = 0.095), central apneas (*p*_MWU_ = 0.512, *p*_A_ = 0.040), mixed apneas (*p*_MWU_ = 0.072, *p*_A_ = 0.049), and hypopneas (*p*_MWU_ = 0.069, *p*_A_ = 0.213) showed a slight trend towards a shorter event duration. In addition, when taking age, BMI, and AHI into account, the AHT% was significantly higher in normotensive females. Previously, longer respiratory events have been linked to an increased risk of all-cause mortality and cardiovascular events [10, 13] but conflicting results have also been reported [14]. The present findings suggest that shorter respiratory events are related to hypertension in females. Furthermore, hypertensive males had higher HT% than their normotensive counterparts but there was no significant difference in respiratory event durations. We speculate that in our present patient population, one explanation for the presence of hypertension can be traced to the natural development of OSA. First, hypertensive patients were significantly older as compared with normotensive patients. Previously, it has been shown that the prevalence of OSA increases with age but starts to decline after the age of 60 years [24]. Furthermore, patients with severe OSA may be at higher risk for fatal cardiovascular events compared with the patients having mild-to-moderate OSA, in whom cardiovascular events may be more often non-fatal in nature [10]. Thus, it could be speculated that if a patient has hypertension and a severe form of OSA (i.e., mainly apneas with long durations), he/she has also a higher probability of suffering from other cardiovascular comorbidities, besides hypertension, possibly leading to death at a younger age. Second, because normotensive patients were much younger than hypertensive patients, it could be speculated that they have had sleep apnea for a shorter period of time which has not been “sufficient” to allow hypertension to develop. However, as we do not have information on how long OSA has been present with each individual, these possible explanations are highly speculative and should be interpreted with caution.

It is known that in patients with severe OSA, sleepy patients have a higher AHI and apnea index compared with non-sleepy patients [25]. In line with this, we found that the patients with AHT% > 45% were subjectively sleepier than the other patients despite the fact that all patients had severe OSA. As the variation in AHI was only capable of explaining 7.7% of the variation in the AHT% among patients with AHT% > 45%, we propose that the link between a high AHT% and subjective sleepiness could stem from both the increased number and the longer duration of respiratory events. Previously, it has been suggested that the primary cause for daytime sleepiness in OSA patients is the fragmented sleep caused by repetitive respiratory event–related arousals [2]. Our present findings support this concept, as we observed that the arousal index and the ESS scores increased significantly in individuals in the higher AHT% categories.

## Limitations

One major limitation of the present study is the relatively small number of female patients. As seen in Table 3, the statistical significance of differences was on the borderline in many of the variables, especially the differences observed in AT% and HT% between normotensive and hypertensive females. However, as the differences were so clearly observable, we believe that our results would have been strengthened had we been able to examine a higher number of females. As a second limitation, the patient population consisted only of patients with severe OSA. We decided to focus only on patients with severe OSA as it is well known that especially severe OSA is an important risk factor for hypertension [26] and that these patients are in the most urgent need of treatment. However, due to this selection, further research is needed to find out whether our findings can be generalized also to mild and moderate OSA patients. A third limitation is the lack of information on other comorbidities except for hypertension nor did we have access to detailed and exact information on the use of antihypertensive and other medications. It is possible that different antihypertensive drugs exert distinctive effects on the durations of respiratory events and this could have a slight effect on the results. Information on other types of cardiovascular diseases could also have assisted in explaining our somewhat unexpected results. As we are convinced that various characteristics of the respiratory events are differently linked to adverse health consequences, further studies are warranted to investigate this topic. Fourth, we did not consider in which sleep stage the individual respiratory events had occurred. We are aware that REM-related OSA is more strongly linked to hypertension than NREM-related OSA [27] and that REM-related OSA is a phenotype more commonly encountered in women [28]. However, since only normotensive males had slightly more REM sleep as compared with hypertensive males, we assume that this limitation does not jeopardize our conclusions. Finally, the role of body posture on the duration of respiratory events was not evaluated; this should be assessed in future investigations.

## Conclusions

The polysomnographic characteristics of severe OSA differ significantly between normotensive and hypertensive patients. Moreover, significant inter- and intra-gender variations exist in the percentage of total apnea and hypopnea time between hypertensive and normotensive patients with severe OSA. As hypertensive patients were older than the normotensive patients regardless of gender, hypertension could be related to the development of OSA or to the time that the patient has had the disease. Thus, early detection of OSA would be highly beneficial, not only preventing the development of hypertension but potentially also in combating the other severe OSA-related comorbidities.
